# Correlated expression of the body, face, and voice during character portrayal in actors

**DOI:** 10.1038/s41598-022-12184-7

**Published:** 2022-05-18

**Authors:** Matthew Berry, Sarah Lewin, Steven Brown

**Affiliations:** Department of Psychology, Neuroscience & Behaviour, McMaster University, 1280 Main St. West, Hamilton, ON L8S 4K1 Canada

**Keywords:** Psychology, Human behaviour

## Abstract

Actors are required to engage in multimodal modulations of their body, face, and voice in order to create a holistic portrayal of a character during performance. We present here the first trimodal analysis, to our knowledge, of the process of character portrayal in professional actors. The actors portrayed a series of stock characters (e.g., king, bully) that were organized according to a predictive scheme based on the two orthogonal personality dimensions of assertiveness and cooperativeness. We used 3D motion capture technology to analyze the relative expansion/contraction of 6 body segments across the head, torso, arms, and hands. We compared this with previous results for these portrayals for 4 segments of facial expression and the vocal parameters of pitch and loudness. The results demonstrated significant cross-modal correlations for character assertiveness (but not cooperativeness), as manifested collectively in a straightening of the head and torso, expansion of the arms and hands, lowering of the jaw, and a rise in vocal pitch and loudness. These results demonstrate what communication theorists refer to as “multichannel reinforcement”. We discuss this reinforcement in light of both acting theories and theories of human communication more generally.

## Introduction

Human communication is an inherently multimodal process^1–5^. However, most research on communication and emotional expression has looked at single channels alone, such as speech prosody, facial expression, or bodily expression. Perhaps the strongest precedent for cross-modal analysis is in the study of co-speech gesturing. McNeill^6^ has argued that both speech and the gesturing that accompanies it emanate from common “growth points” that create parallel and mutually reinforcing routes of expression. While such parallelism relates to the semantic aspect of communication, beating gestures of the hands and head are a mechanism for gesturally marking points of phonological stress in the speech stream^7, 8^.

Birdwhistell^9, 10^ developed a micro-level analysis of the body movements that accompany speech, as formalized into the study of what he called kinesics (see also Dael et al., 2016)^11^. He examined these movements as a series of isolable yet combinable “kinemes”, each one having an intensity, extent, and duration. A basic tenet of Birdwhistell’s analysis was that the various expressive modalities operate through a process of *multichannel* *reinforcement* and thus redundancy. Such redundancy “makes the contents of messages available to a greater portion of the population than would be possible if only one modality were utilized to teach, learn, store, transmit, or structure experience” (Birdwhistell,^10^:107). Outside of the realm of speech, there has been a small amount of cross-modal work for music. For example, high-pitched vocal sounds are accompanied by a raising of the brow and a lowering of the jaw^12, 13^. A primary aim of the present study is to look beyond analyzing pairs of modalities and to investigate the *trimodal* relationship between the voice, face, and body during acts of communication, with a focus on character portrayal during acting.

However, the most neglected topic in unimodal research has been that of the bodily expression of emotion. While numerous studies have examined the perception of expression—in either static^14^ or dynamic presentations^15, 16^—much less work has focused on the production process itself. It is notable that, in the context of the current study on acting, many studies of bodily expression have employed trained actors to “act out” exemplars of the emotions under investigation, although the focus has never been on the process of acting itself, but rather on the perception of emotions (e.g., Gross et al., Volkova et al.)^17, 18^. For example, Wallbott^19^ examined the bodily expression of 14 common emotions. Body movements of the head and upper limbs were coded in a free format based on video analysis. The results showed that high-intensity emotions like hot anger and joy were characterized by expansive movements of the upper limbs. Dael, Mortillaro, and Scherer^20^ performed a detailed analysis of body-wide expressions for 12 emotions, as produced by actors. Three of the major dimensions of bodily expression observed in this analysis were the location of the arms in relation to the body, the positioning of the upper body relative to the lower body, and the positioning of the head relative to the body. Van Dyck et al.^21^ examined the impact of happy vs. sad mood induction on the body movements produced during an improvised dance to neutral music. The results showed that happy induction, compared to sad, led to more-expansive movements of the hands and arms, as well as increases in the velocity and acceleration of the limb movements.

In the closest precursor to the current work, Scherer and Ellgring^3^ carried out one of the few studies of multimodal integration by comparing vocal, facial, and bodily expressions of emotion (where the latter were based on the body data from Wallbott)^19^. Contrary to providing support for a strong model of multichannel reinforcement, Scherer and Ellgring found that “individuals tend to use only part of the possible expressive elements in the vocal, gestural, and facial display of an emotion” and that “nonverbal elements combine in a logical ‘or’ instead of a logical ‘and’ association” (p. 169).

The current study looks beyond everyday communication and expression to examine the skills that professional actors bring to the portrayal of fictional characters and their emotions. Historically, acting theories have been polarized along the lines of whether actors engage in either a psychological process of identification with their characters (the “inside-out” approach) or instead an externalization of the gestural features of the characters being portrayed (the “outside-in” approach)^22–26^. While both methods are pervasive in contemporary actor training^27–29^, the ability of researchers to study actors’ internal mental states experimentally presents significant obstacles. Therefore, behavioral work on acting has thus far focused on the external manifestations of character portrayal—in other words, on the “gestural codes” used by actors to create portrayals of characters and their emotions—rather than on actors’ internal psychological states.

**Figure 1:**
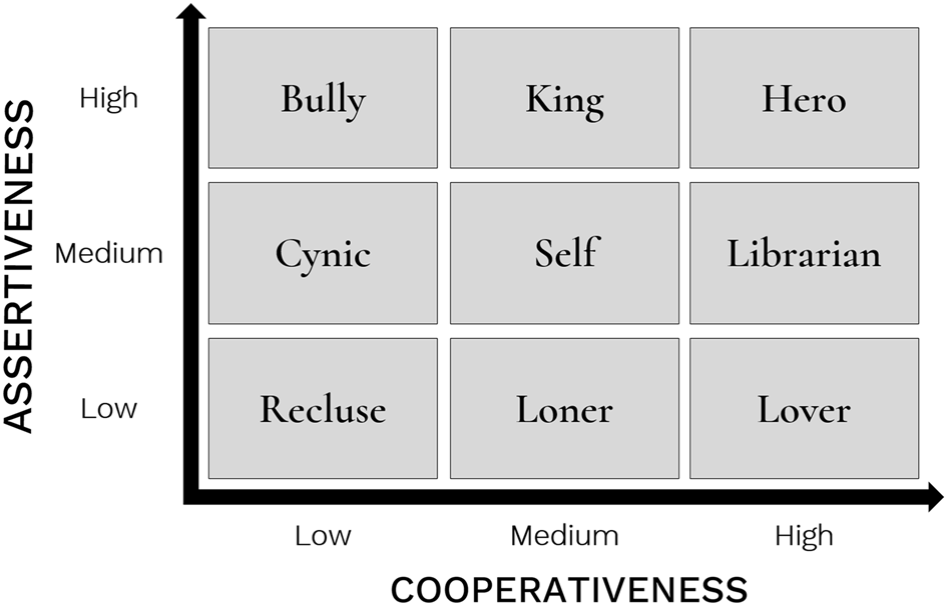
Character scheme. The figure shows the 9 stock characters used in the study, as organized into a 3 × 3 scheme based on a crossing of 3 levels of assertiveness and cooperativeness, respectively. The scheme is adapted from Berry and Brown^23^.

Our approach to quantifying the gestural basis of acting has been predicated on the development of a dimensional scheme for classifying literary characters. In Berry and Brown^30^, we presented a proposal for a systematic classification of characters based on personality dimensions, using a modification of the Thomas-Kilmann Conflict Mode Instrument^31–33^, which classifies personality along the two orthogonal dimensions of *assertiveness* and *cooperativeness*. We conducted a character-rating study in which participants rated 40 stock characters with respect to their assertiveness and cooperativeness. The results demonstrated that these ratings were orthogonal. The scheme is shown in Fig. 1, in which a crossing of 3 levels of assertiveness and 3 levels of cooperativeness results in 9 character types. We selected single exemplars of these 9 types for the acting experiment, as shown in Fig. 1. In two previous studies of acting, we examined the vocal and facial correlates, respectively, of acting as professional actors performed portrayals of the 9 characters shown in the figure. The results demonstrated significant effects of character assertiveness on vocal pitch and loudness^23^, as well as significant effects of character cooperativeness on the expansion of facial segments for the brow, eyebrow, and lips^34^. Hence, the results revealed that different expressive modalities are specialized in conveying information related to a character’s assertiveness and cooperativeness, respectively. This might be consistent with the claim of Scherer and Ellgring^3^ that not all expressive channels are used equally. An additional finding of the face study was that there was a parallel effect of character assertiveness to the emotion dimension of arousal, as well as a parallel effect of character cooperativeness to the emotion dimension of valence. Such character/emotion parallels will be revisited in the current analysis of bodily expression.

The principal objective of the present study was to extend our previous work on the voice and face in order to quantify the bodily correlates of character portrayal for the first time, to the best of our knowledge. We did this in a production study using professional actors and a high-resolution 3D motion capture set-up in a black-box performance laboratory. A group of 24 actors performed a semantically-neutral script while portraying 8 stock characters and the self (see Fig. 1). As with our previous study of the facial correlates of acting^34^, we chose not to examine single motion-capture markers in isolation, but instead to look at two-marker *segments* so as to analyze the relative expansion/contraction of these segments. Segments define not only fundamental units of bodily expression but also the *shapes* associated with expression, as revealed in seminal work by Laban on the shapes of body movement^35^. In particular, we analyzed six segments: two related to posture and four related to bilateral movement of the upper limbs. All of them are contained within the Body Action and Posture coding system^20, 36^, and are associated with distinct components of expressive body movement. The postural segments were head flexion/extension and torso flexion/extension, both in the sagittal plane. The four limb segments were horizontal and vertical movements of both the arms and hands bilaterally. Expansive movements in all six segments have been shown to be associated with high-arousal and/or positive valenced emotions^3, 19–21^, for example, the lifted head of pride or the raised arms of the victory pose. In addition, there are numerous precedents for using these same segments in previous segmental analyses of body movement^18, 37–50^.

The second major objective of the current study was to take advantage of our previous work in order to examine correlated expression across the body, face, and voice so as to provide a holistic trimodal account of the gestural correlates of acting. Our previous study revealed bimodal correlations between jaw lowering in the face and rises in both pitch and loudness in the voice, all of which were associated with the assertiveness of characters^34^. These correlations between the face and voice serve as the best foundation to build upon in developing a trimodal account of acting. Therefore, we hoped to discover bodily correlates of character assertiveness that could be added onto them. Overall, our major predictions are that 1) the six selected segments will provide adequate variance across characters and emotions to generate informative profiles, 2) high-assertive characters—akin to high-intensity emotions^3, 19, 34^—will demonstrate expansion of the limb and postural segments, and 3) the dimension of character assertiveness will provide the strongest evidence for a trimodal relationship among effectors during acting.

## Methods

### Participants

**Figure 2:**
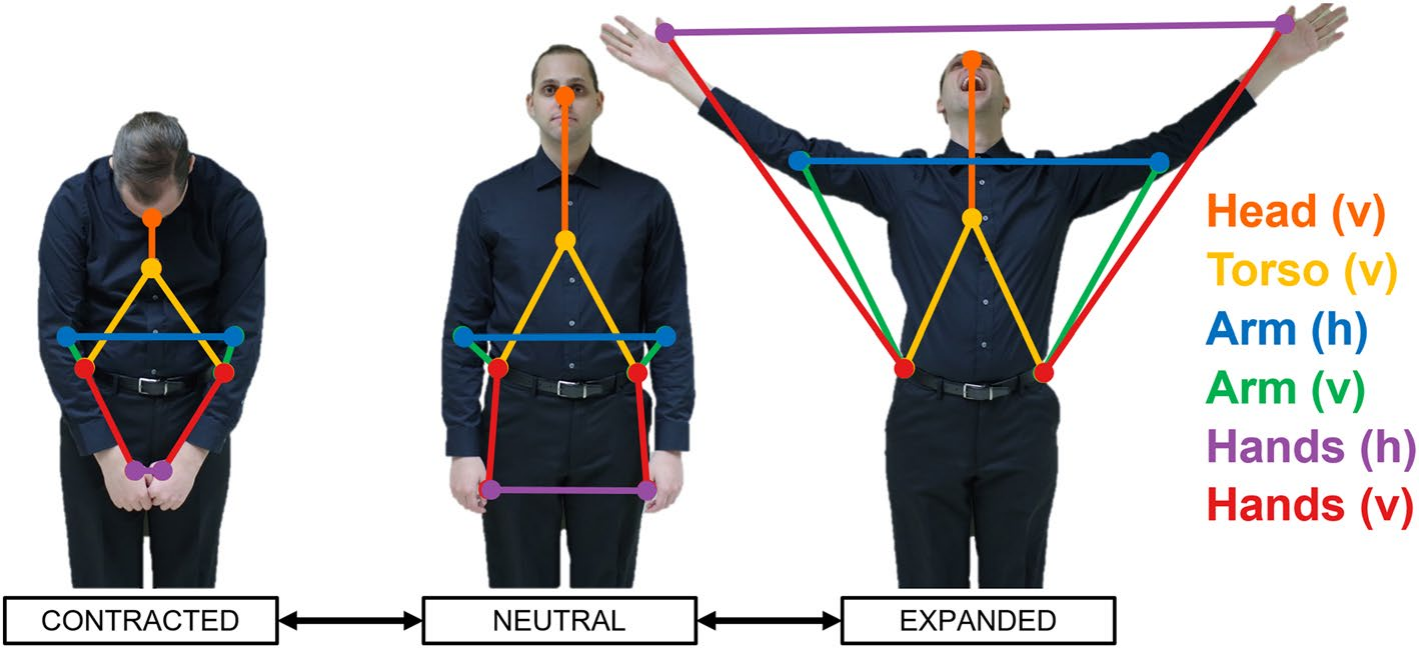
The six body segments. The figure shows a visual representation of the six body segments analyzed in the study, as related to expansion/contraction of the head, torso, arms, and hands, where “(v)” designates vertical movement and “(h)” horizontal movement. The photos are courtesy of author MB. The model gave consent for the use of these photographs.

Twenty-four actors (14 males, 10 females; M_Age_ = 42.5 ± 14 years) were recruited for the experiment through local theatre companies and academic theatre programs. All actors were legal adults who spoke English either as their native language or fluently as their second language (n = 1). Actors were selected for their overall level of acting experience (i.e., a minimum of three years of experience; M_Exp_ = 27.5 ± 14.3). Fourteen held degrees in acting, and two were pursuing degrees in acting at the time of the experiment. Seventeen of the 24 participants self-identified as professional actors. All participants gave written informed consent and were given monetary compensation for their participation. In addition, written informed consent was provided by the model used in Fig. 2 and Supplementary Fig. 1 for publication of identifying information/images in an online open-access publication. The study was approved by the McMaster University Research Ethics Board, and all experiments were conducted in accordance with relevant guidelines and regulations.

### Characters and emotions

The methods and procedures are similar to those reported in Berry and Brown (2019, 2021)^23, 34^. The actors performed the 9 characters from the 3 × 3 (assertiveness x cooperativeness) classification scheme validated by Berry and Brown^30^ (see Fig. 1). The actors, in addition to portraying characters, performed 8 basic emotions (happy, sad, angry, surprised, proud, calm, fearful, and disgusted) and neutral, as based on previous emotion studies using actors^3, 19, 51–53^. The selected emotions were grouped according to an approximate dimensional analysis (e.g., Russell, 1980)^54^, rather than examining them individually (see also Castellano et al., 2007)^15^. A 2 × 2 scheme was used according to the valence and arousal of each emotion as follows: positive valence + high arousal (happy, proud, surprised), negative valence + high arousal (angry, fearful, disgusted), positive valence + low arousal (calm), and negative valence + low arousal (sad). The order of presentation of the 9 characters and 9 emotions was randomized across the 18 trials for each participant. The actors performed a semantically neutral monologue-script for each of the 18 trials. The script was created for the study and consisted of 7 neutral sentences (M = 6 ± 1.4 words/sentence) derived from a set of 10 validated linguistically-neutral sentences from Ben-David et al.^55^. Each trial lasted approximately two minutes, and the full set of trials lasted no more than 45 min. At the end of the session, the actor was debriefed and compensated.

### Motion capture

The experiment took place in a black-box performance laboratory. Actors performed each of the 18 trials on stage, facing an empty audience section. The performances were video- and audio-recorded using a Sony XDCam model PXW-X70. A Qualisys three-dimensional (3D) passive motion-capture system was used to record body gestures and facial expressions for each actor. Sixteen Qualisys Oqus 7 infrared cameras captured marker movement in three dimensions at a sampling rate of 120 Hz^56, 57^. Participants were equipped with 61 passive markers placed on key body/facial landmarks, providing bilateral full-body coverage. Of these, 37 markers were placed on the torso and limbs, 4 on the head via a cap, and 20 markers on the face. The markers used in the present analysis were placed bilaterally on the thumbs, elbows, and hips, as well as two single midline markers placed on the sternum and bridge of the nose, respectively.

### Data processing and cleaning

Marker movements for the body were recorded in 2D and reconstructed in 3D for analysis. The 2D-tracked motion data were processed using the Qualisys reconstruction algorithm, creating an analyzable 3D model within the user interface (UI; Qualisys, 2006)^57^ . Following this, each trial was cleaned manually using the 3D model via the UI (i.e., each marker and trajectory was identified manually, provided a label, and extracted). Extraneous trajectories (e.g., noise, errors, reflective artifacts, unassigned or outlying markers) were excluded. No interpolation was done (i.e., no gaps in the 3D motion trajectory were filled). Instead, the data from a particular marker were temporally omitted. This was done to prevent the system from incorrectly interpolating and/or skewing the motion data and thereby artificially changing the mean. The cleaned X coordinates (anterior–posterior movement), Y coordinates (right-left movement), and Z coordinates (superior-inferior movement) were extracted into data tables for further analysis.

### Transformation of variable parameters

The variables of interest in this study are those related to expansion and contraction of body segments. From the 61 available markers, we selected a subset of 8 for the current analysis: markers located on the nose bridge, sternum, left and right elbow, left and right thumb, and left and right hip. Pairs of markers were combined into 6 body segments whose expansion and contraction were measured in three dimensional space, as shown in Fig. 2. We examined two vertical postural segments: (1) “head”, extending from the sternum to the bridge of the nose, to indicate sagittal flexion/extension of the head; and (2) “torso”, extending from the left/right hip to the sternum, to indicate sagittal flexion/extension of the torso at the waist. Next, we examined four segments related to horizontal and vertical expansion/contraction of the upper limbs: (3) “horizontal arm”, extending between the left and right elbows; (4) “vertical arm”, extending from the left/right hip to the left/right elbow; (5) “horizontal hand”, extending between the left and right thumbs; and (6) “vertical hand”, extending from the left/right hip to the left/right thumb. The term “left/right” implies that the mean was taken for the two sides of the body for that segment. Each segment’s length was calculated from the raw exported X, Y, and Z coordinates for the pair of contributing markers using the following formula for Euclidean distance:

$${\text{d }} = \, \surd \left( {{\text{x}}_{{2}} {-}{\text{ x}}_{{1}} } \right)^{{2}} + \, \left( {{\text{y}}_{{2}} {-}{\text{ y}}_{{1}} } \right)^{{2}} + \, \left( {{\text{z}}_{{2}} {-}{\text{ z}}_{{1}} } \right)^{{2}}$$

where d is the Euclidean distance (i.e., the absolute geometric distance) between two points in 3D space, and x, y, and z are the 3D coordinates of a single sample at time (2) and time (1), respectively. A time series of the Euclidean distance for each body segment was created for each approximately-2-min trial. The mean segment length across this time series was calculated for each of the 6 body segments using the following formula:

$${\text{M}}_{{\text{d ij}}} = \sum {\text{ d}}_{{{\text{ij}}}} /{\text{ sr}}*\left( {{\text{t}}_{{{\text{ij}}}} } \right)$$

where M_d_ is the mean Euclidean distance in mm between marker pairs over the length of the entire trial (i.e., the mean segment length), d is the segment length, sr is the motion capture sample rate (i.e., 120 Hz), and t is the time in seconds of the entire trial. This resulted in a total of 6 parameters for the analysis (i.e., 6 body-segment means). Each body-segment parameter mean was extracted for each participant (i) for each character or emotion condition (j).

### Correcting for body-size differences

A “percent change” transformation was applied to the 6 segmental parameter means in order to eliminate any bias caused by subject-related differences in body size. This was carried out by subtracting the mean segmental lengths for the neutral emotion condition (i.e., performing the script devoid of any character or emotion) from the means for each character and emotion trial, as per the following formula:

$$\% {\text{ change }} = { 1}00 \, * \, \left( {\left( {{\text{M}}_{{{\text{d}}[{\text{performance}}]}} {-}{\text{ M}}_{{{\text{d}}[{\text{neutral}}]}} } \right) \, /{\text{ M}}_{{{\text{d}}[{\text{neutral}}]}} } \right)$$

where the percent change is the difference between the mean Euclidean distance for a participant’s given performance condition (character or emotion) and the participant’s neutral emotion condition, scaled to the neutral condition, and then multiplied by 100. As a result, all data for the characters and emotions are reported as a percent change relative to the neutral emotion condition. Following this transformation, each parameter was visually screened for extreme outliers, of which none were found. Finally, to reduce handedness effects, the bilateral average was taken for the vertical arm, vertical hand, and torso.

### Analysis of variance

Statistics were conducted in R 4.0.2 (R Core Team, 2018)^58^ . Each of the 6 transformed parameters was analyzed using a two-way repeated-measures analysis of variance (RM ANOVA), which fits a linear model (lm) using the *stats* package (v3.6.2, R Core Team, 2013)^59^. For the character trials, the two orthogonal dimensions of assertiveness and cooperativeness were treated as fixed effects (i.e., within-subject factors), while subject was treated as the random effect (i.e., error). For the emotion trials, the two approximated dimensions of valence and arousal from the circumplex model of emotion^54^ were treated as fixed effects, while subject was again treated as the random effect. The neutral emotion condition—which was used as the baseline condition for data normalization—was not included in either of these analyses. The final sample for the repeated-measures ANOVA’s was therefore n = 216 for characters (9 characters × 24 participants) and n = 192 for emotions (8 emotions × 24 participants). Statistical significance levels were set to α < 0.05, and adjustments for repeated testing for the group of 6 segmental parameters were made using Bonferroni corrections (i.e., α/6 for each segment, resulting in a corrected threshold of α < 0.008)^23, 60^. The significance of statistical analyses and the estimates of effect size using general eta-squared (ƞ^2^) and partial eta-squared (ƞ_p_^2^) were calculated using the *rstatix* package (v0.7.0, Kassambara, 2019)^61^.

### Cross-modal correlation analysis

We used the combination of the character and emotion trials with the *stats* package (v3.6.2, R Core Team, 2013)^59^ to calculate Pearson product-moment correlations between the 6 body segmental parameters, 4 facial segmental parameters for the brow, eyebrows, lips, and jaw (reported in Berry & Brown, 2021)^34^, and the 2 vocal parameters of pitch (in cents) and loudness (in decibels) (reported in Berry & Brown)^23^. All parameters were z-score transformed within-subject in order to avoid any scale-related artifacts due to intermodal variability in comparing parameters across the body, face, and voice. The neutral emotion condition—which was used as the baseline condition for data normalization—was not included in this analysis. The final sample for the cross-modal correlations was therefore n = 408 (9 characters × 24 participants + 8 emotions × 24 participants). Statistical significance was set to α < 0.05, and adjustment for repeated testing of 53 analyzed intermodal correlations was made using Bonferroni corrections (i.e., α/53 for each correlation, resulting in a corrected threshold of α < 0.0009). An additional 13 intramodal correlations are presented in Table 3 (e.g., the correlation between the lips and brow within the face), but these were not correlations of interest, only the 53 intermodal correlations.

## Results

### Segmental body analysis

**Figure 3:**
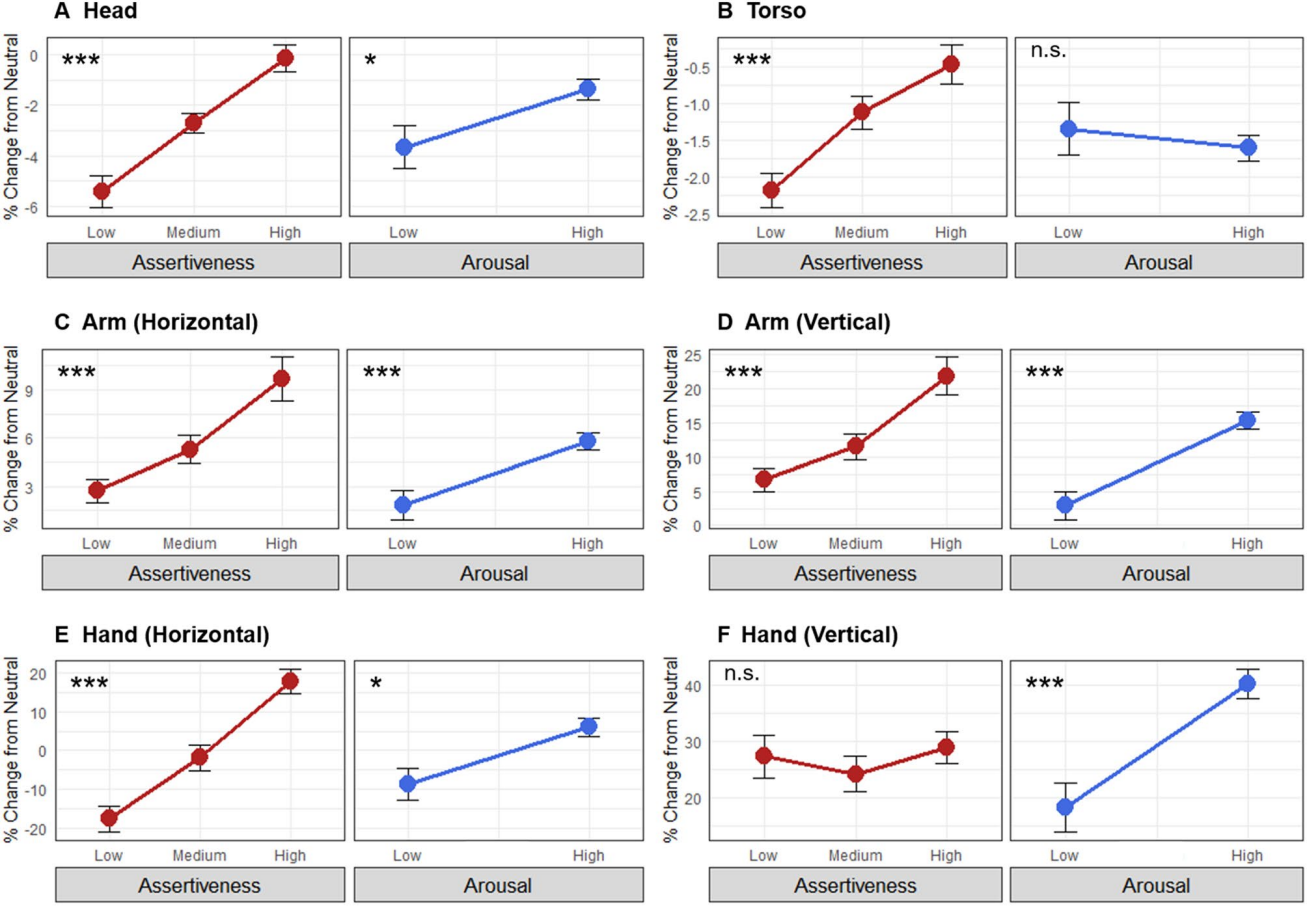
Body effects of character assertiveness and emotional arousal. The figure shows the effect of character assertiveness (left panels in red) and emotional arousal (right panels in blue) on the parameter means of the 6 body segments. Values for each segment are the percent change relative to the neutral emotion condition, which corrects for the diversity of body dimensions across participants. Error bars indicate the standard error of the mean. Significance values are from a repeated-measures ANOVA regression model for the main effects of character dimensions. *p < .05 and ***p < .001 after Bonferroni correction, n.s., not significant. See Table 1 for the full character descriptions and Table 2 for the full emotion descriptions.

The first set of analyses examined the bodily correlates of both character portrayal and emotional expression in actors. Table 1 provides the full RM ANOVA results for the 6 segment means across the 9 characters, as grouped according to assertiveness and cooperativeness. Figure 3 reveals that there were significant and monotonic effects of character assertiveness on body expansion for both the horizontal and vertical dimensions of arm movement, as well as for horizontal expansion of the hand segment, but not vertical raising of the hands. Additional effects were seen for the head and torso such that increased character assertiveness was associated with a raising of the head and a straightening of the torso. Supplementary Fig. 1 presents photographs comparing the average limb positions for high assertiveness with the neutral posture.

**Table 1:** Repeated-measured ANOVA results for the character dimensions.

Segment	Effect	DFn	DFd	SSn	SSd	*F*	*p*	sig	*η*^2^	*η*_p_^2^
Head	Assert	2	46	1011.25	707.33	32.883	** < 0.001**	***	0.26	0.59
	Coop	2	46	191.74	631.69	6.981	**0.002**	**	0.06	0.23
	Assert:Coop	4	92	375.80	1515.23	5.704	** < 0.001**	***	0.12	0.20
Torso	Assert	2	46	107.55	112.71	21.949	** < 0.001**	***	0.14	0.49
	Coop	2	46	73.77	198.72	8.539	** < 0.001**	***	0.10	0.27
	Assert:Coop	4	92	16.12	336.40	1.102	0.360	n.s	0.02	0.05
Arm (h)	Assert	2	46	1782.09	2557.66	16.026	** < 0.001**	***	0.15	0.41
	Coop	2	46	764.40	1842.60	9.542	** < 0.001**	***	0.07	0.29
	Assert:Coop	4	92	1527.07	6001.13	5.853	** < 0.001**	***	0.13	0.20
Arm (v)	Assert	2	46	8661.44	11,384.28	17.499	** < 0.001**	***	0.15	0.43
	Coop	2	46	4335.08	10,938.16	9.116	** < 0.001**	***	0.08	0.28
	Assert:Coop	4	92	6338.90	25,960.47	5.616	** < 0.001**	***	0.12	0.20
Hand (h)	Assert	2	46	45,450.52	38,332.85	27.271	** < 0.001**	***	0.29	0.54
	Coop	2	46	4050.18	21,938.31	4.246	*0.020*	*	0.04	0.16
	Assert:Coop	4	92	19,486.96	51,185.02	8.756	** < 0.001**	***	0.15	0.28
Hand (v)	Assert	2	46	833.85	22,142.38	0.866	0.427	n.s	0.01	0.04
	Coop	2	46	8854.43	32,626.03	6.242	**0.004**	**	0.07	0.21
	Assert:Coop	4	92	6346.17	55,927.92	2.610	*0.041*	*	0.05	0.10

Summary of the two-way repeated-measures ANOVA for each segment, after controlling for body diversity using the neutral emotion condition. Measures of effect size include general eta squared (η^2^) and partial eta squared (η_p_^2^). *P* values that failed to reach significance after Bonferroni corrections are in *italics*. *P* values that retained significance after Bonferroni corrections are in bold. *p < .05, **p < .01, ***p < .001, n.s., not significant. Abbreviations: Assert, assertiveness; Coop, cooperativeness; DFd, denominator degrees of freedom; DFn, numerator degrees of freedom); (h), horizontal; ges, general eta squared; n.s., not significant; pes, partial eta squared; sig, significance level; SSd, sum of squares denominator; SSn, sum of squares numerator; (v), vertical.

**Figure 4:**
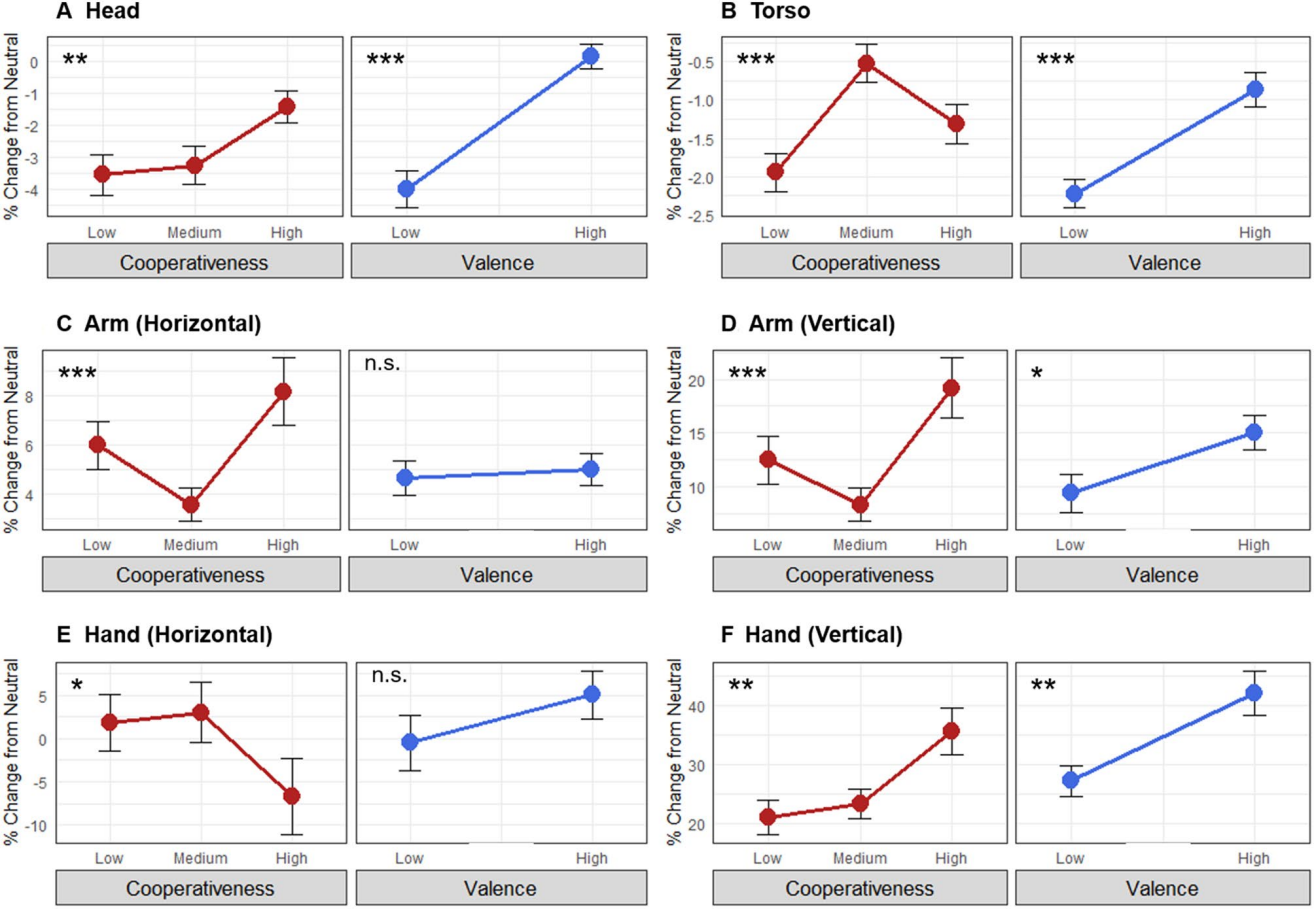
Body effects of character cooperativeness and emotional valence*.* The figure shows the effect of character cooperativeness (left panels in red) and emotional valence (right panels in blue) on the parameter means of the 6 body segments. Values for each segment are the percent change relative to the neutral emotion condition, which corrects for the diversity of body dimensions across participants. Error bars indicate the standard error of the mean. Significance values are from a liner mixed-effects regression model for the main effects of character dimensions. *p < .05, **p < .01, and ***p < .001 after Bonferroni correction, n.s., not significant. See Table 1 for the full character descriptions and Table 2 for the full emotion descriptions.

Table 2 provides a full summary of the RM ANOVA conducted for the 6 segment means across the 8 emotions. The emotions are grouped according to the two emotion dimensions of arousal and valence, where the arousal results are shown in the right panels of Fig. 3 and the valence results in the right panels of Fig. 4. The results for emotional arousal strongly mirrored those for character assertiveness, with two exceptions. The first was for the torso segment, which showed a non-significant effect of arousal (*F*(1,23) = 0.361, *p* = 0.554) compared to a monotonic expansion for assertiveness (*F*(2,46) = 29.949, *p* < 0.001). The second was for the vertical hand segment, which showed increased raising of the hand with increasing arousal (*F*(1,23) = 22.741, *p* < 0.001), compared to a null effect of assertiveness (*F*(2,46) = 0.866, *p* = 0.427). Overall, there was a similar trend toward increasing body expansiveness with increases in both character assertiveness and emotional arousal.

**Table 2:** Repeated-measures ANOVA results for the emotion dimensions.

Segment	Effect	DFn	DFd	SSn	SSd	*F*	*p*	sig	*η*^2^	*η*_p_^2^
Head	Arousal	1	23	189.69	569.39	7.662	*0.011*	*	0.12	0.25
	Valence	1	23	833.83	527.69	36.344	** < 0.001**	***	0.37	0.61
	Arousal:Valence	1	23	149.21	325.77	10.534	**0.004**	**	0.10	0.31
Torso	Arousal	1	23	2.43	154.83	0.361	0.554	n.s	0.01	0.02
	Valence	1	23	89.38	108.65	18.920	** < 0.001**	***	0.22	0.45
	Arousal:Valence	1	23	2.81	59.76	1.083	0.309	n.s	0.01	0.05
Arm (h)	Arousal	1	23	578.83	908.54	14.653	** < 0.001**	***	0.20	0.39
	Valence	1	23	5.44	724.38	0.173	0.681	n.s	0.00	0.01
	Arousal:Valence	1	23	98.47	720.91	3.142	0.090	n.s	0.04	0.12
Arm (v)	Arousal	1	23	5568.45	5284.06	24.238	** < 0.001**	***	0.28	0.51
	Valence	1	23	1557.64	5775.32	6.203	*0.020*	*	0.10	0.21
	Arousal:Valence	1	23	34.56	3156.64	0.252	0.621	n.s	0.00	0.01
Hand (h)	Arousal	1	23	7914.88	27,735.07	6.564	*0.017*	*	0.14	0.22
	Valence	1	23	1488.17	14,254.86	2.401	0.135	n.s	0.03	0.10
	Arousal:Valence	1	23	346.92	7884.56	1.012	0.325	n.s	0.01	0.04
Hand (v)	Arousal	1	23	17,270.78	17,467.60	22.741	** < 0.001**	***	0.21	0.50
	Valence	1	23	10,564.16	22,836.68	10.640	**0.003**	**	0.14	0.32
	Arousal:Valence	1	23	23.69	26,385.80	0.021	0.887	n.s	0.00	0.00

Summary of the two-way repeated measures ANOVA for each segment, after controlling for body diversity using the neutral emotion condition. Measures of effect size include general eta squared (η^2^) and partial eta squared (η_p_^2^). *P* values that failed to reach significance after Bonferroni corrections are in *italics*. *P* values that retained significance after Bonferroni corrections are in bold***.*** *p < .05, **p < .01, ***p < .001, n.s., not significant. Abbreviations: DFd, denominator degrees of freedom; DFn, numerator degrees of freedom; (h), horizontal; ges, general eta squared; n.s., not significant; pes, partial eta squared); sig, significance level; SSd, sum of squares denominator; SSn, sum of squares numerator; (v), vertical.

Figure 4 presents the bodily correlates of character cooperativeness and the related emotion dimension of valence. The results for cooperativeness demonstrated significant but fewer monotonic patterns compared to those for character assertiveness. Significant and monotonic effects were only seen for the head and the vertical segment of the hand. These results were mirrored by the results for emotional valence, with additional expansive effects seen with the head and torso. Overall, the body analysis demonstrated a far stronger and more linear effect for character assertiveness (and emotional arousal) than for character cooperativeness (and emotional valence).

### Cross-modal correlations

**Figure 5:**
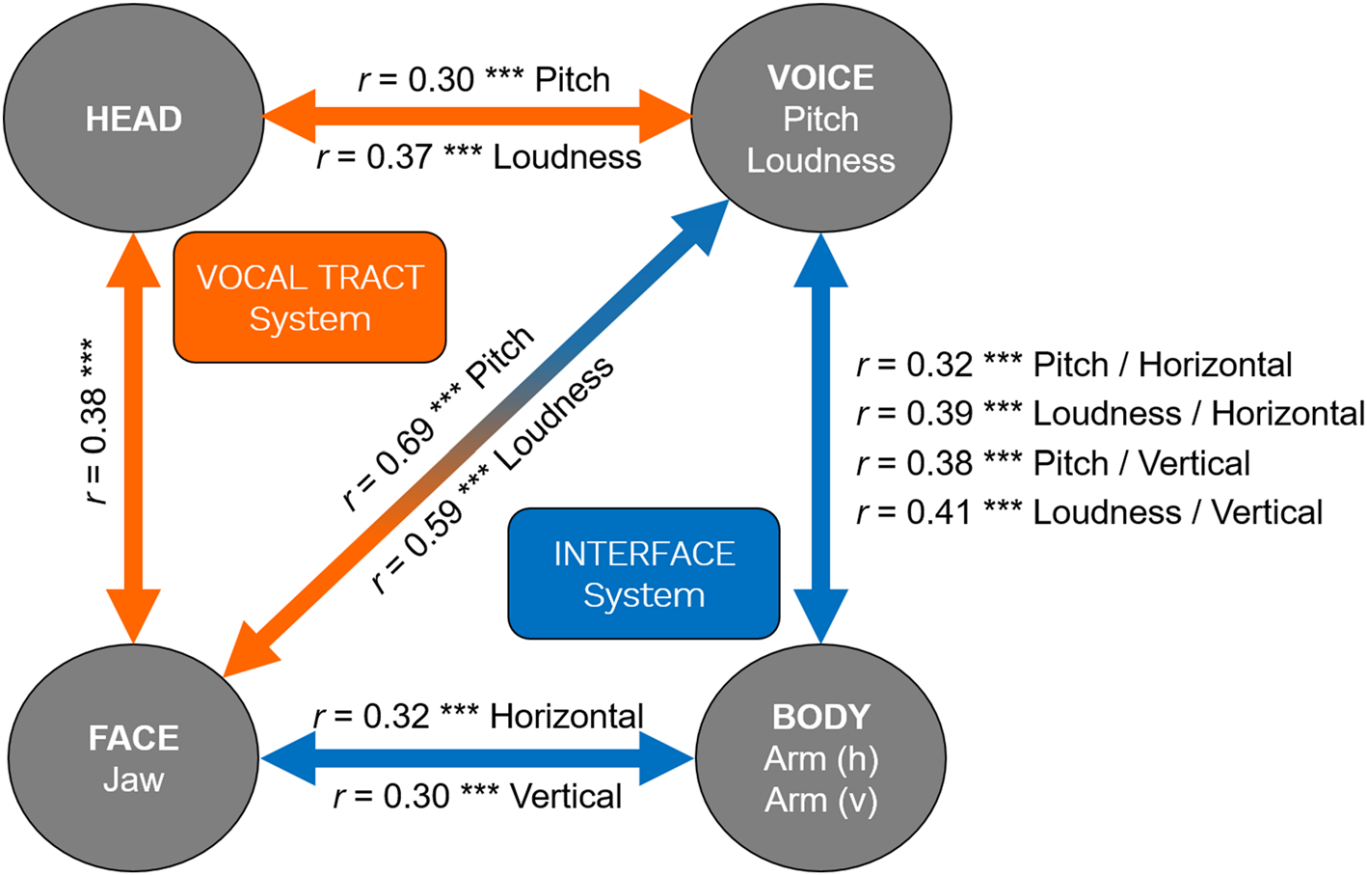
A summary of the major cross-modal correlations*.* The figure shows the combined correlations of the character and emotion conditions for the parameter means for the head (head raising), voice (increases in pitch and loudness), face (jaw lowering), and body (horizontal and vertical arm expansion). Orange lines represent significant correlations between parameters related to the “vocal tract system”. Blue lines represent significant correlations between parameters related to the “interface system”. Pearson product-moment correlation values are given for each correlation pairing. *** *p*_*CORR*_ < .001. See Table 3 for full character and emotion correlations. Note: Pearson product-moment correlations between the Head and Body are *r* = 0.17 * for vertical arm raising and *r* = 0.15 n.s. for horizontal arm expansion, respectively. Abbreviations: (h), horizontal; (v), vertical.

In order to examine the cross-modal relationship between the current body results and our previous results for vocal prosody^23^ and facial expression^34^, we examined pairwise correlations between all of the relevant parameters, and corrected for multiple comparisons using a Bonferroni correction. The correlation table is shown in Table 3. The individual regression analyses are shown in Supplementary Figs. 2–4. A summary of the key significant findings is presented in Fig. 5. These are presented as two triadic relationships. The first one is a “vocal tract system” made up of the voice, jaw, and head. Significant pairwise correlations were seen among all three effectors, with the strongest values observed between jaw lowering and vocal pitch height and loudness. The vocal tract system reflects a coupling of effectors required for efficient vocal production. The second triad is that between the voice, face, and body, what is labelled as the “interface system”. Significant correlations were seen for all three sets of pairwise comparisons. Of these, the strongest correlations were again those between the jaw and voice. This was followed by limb/voice correlations between the voice and both the vertical and horizontal segments of arm movement. The weakest relationship, but still significant after correction, was that between jaw movement and arm movement. Outside of the jaw, the correlations were weak between the face and body (see Table 3). Cross-modal correlations with the body were also significant when examining the hands (instead of the arms), but these are not included in Fig. 5 due to the less consistent effect of the hands in the repeated-measures ANOVA analysis for assertiveness.

**Table 3:** Cross-modal correlations.

		VOICE		FACE				BODY					
		Pitch	Loudness	Brow	Eyebrow	Lips	Jaw	Head	Torso	Arm (h)	Arm (v)	Hand (h)	Hand (v)
VOICE	Pitch	NA	** < 0.001**	*0.009*	0.803	** < 0.001**	** < 0.001**	** < 0.001**	0.360	** < 0.001**	** < 0.001**	** < 0.001**	** < 0.001**
	Loudness	0.79***	NA	*0.010*	0.866	*0.010*	** < 0.001**	** < 0.001**	0.236	** < 0.001**	** < 0.001**	** < 0.001**	** < 0.001**
FACE	Brow	0.13	0.13	NA	** < 0.001**	** < 0.001**	** < 0.001**	** < 0.001**	** < 0.001**	0.781	0.144	0.737	** < 0.001**
	Eyebrow	−0.01	0.01	0.62***	NA	** < 0.001**	*0.002*	** < 0.001**	** < 0.001**	0.482	0.872	0.745	** < 0.001**
	Lips	0.19**	0.13	0.37***	0.42***	NA	0.933	** < 0.001**	** < 0.001**	0.309	*0.015*	0.256	*0.031*
	Jaw	0.69***	0.59***	0.24***	0.16	0	NA	** < 0.001**	*0.048*	** < 0.001**	** < 0.001**	** < 0.001**	** < 0.001**
BODY	Head	0.30***	0.37***	0.20**	0.25***	0.25***	0.38***	NA	*0.019*	*0.002*	** < 0.001**	** < 0.001**	** < 0.001**
	Torso	−0.05	0.06	0.22***	0.20**	0.27***	−0.1	0.12	NA	*0.033*	** < 0.001**	*0.004*	** < 0.001**
	Arm (h)	0.32***	0.39***	0.01	−0.03	0.05	0.32***	0.15	0.11	NA	** < 0.001**	** < 0.001**	** < 0.001**
	Arm (v)	0.38***	0.41***	0.07	0.01	0.12	0.30***	0.17*	0.18*	0.78***	NA	** < 0.001**	** < 0.001**
	Hand (h)	0.31***	0.49***	0.02	−0.02	0.06	0.20**	0.18*	0.14	0.26***	0.37***	NA	0.970
	Hand (v)	0.42***	0.30***	0.20**	0.17*	0.11	0.50***	0.18*	−0.19**	0.24***	0.30***	0	NA

Summary of the Pearson product-moment correlations and their significance for each modality: voice (pitch and loudness), face (brow, eyebrow, lips, and jaw), and body (head, torso, horizontal arm, vertical arm, horizontal hand, and vertical hand). The lower triangle contains Pearson *r* values, whereas the upper triangle contains uncorrected *p* values. For the upper triangle, *p* values that failed to reach significance after Bonferroni correction are in *italics*. *P* values that retained significance after Bonferroni corrections are in bold***.*** For the lower triangle, *p_*CORR*_ < .05, **p_*CORR*_ < .01, ***p_*CORR*_ < .001, where “CORR” reflects the adjusted alpha value after Bonferroni correction for the 53 analyzed intermodal correlations, which are a subset of the 66 correlations shown in the table.

## Discussion

In the present study, we examined the bodily correlates of both character portrayal and emotion expression in trained actors. We then compared the results with analyses of facial expression and vocal prosody for the same trials in order to develop an understanding of cross-modal effects during acting. The results revealed strong and monotonic effects of character assertiveness and the related emotion dimension of arousal on body expansion, but weaker and less linear effects of character cooperativeness and the related emotion dimension of valence. For assertiveness, the effects were most robust for bilateral arm expansions in both the vertical and horizontal dimensions, as well as for head raising and torso straightening. We used these data to examine cross-modal correlations with facial expression and vocal prosody in order to provide a holistic trimodal account of the gestural correlates of acting. We found significant correlations across all three groups of effectors, resulting in two triadic relationships among them (see Fig. 5), combining head raising, jaw lowering, rises in vocal pitch and loudness, and both horizontal and vertical expansion of the arms. To the best of our knowledge, these results provide a first picture of the cross-modal relationships among the body, face, and voice during the portrayal of characters by actors. They also reinforce our previously-described relationship between character dimensions and emotion dimensions^34^, but extend them beyond the face and voice to include bodily expression. The results overall are supportive of Birdwhistell’s^9, 10^ concept of multichannel reinforcement in human communication, regardless of whether this communication occurs in everyday or theatrical contexts. An important advantage of the approach that we took in the present study is that, instead of recruiting actors to create prototypical expressions of particular emotions as stimuli for perception studies, we conducted a group analysis of 24 actors that explicitly examined the process of *producing* these expressions to begin with, including the inter-individual variability inherent in these performances.

Scherer and Ellgring’s^3^ study of emotion mentioned in the Introduction—which employed the body data of Wallbott^19^—found a similar multimodal cluster to the one that we found. They labelled it as the “multimodal agitation” cluster, reflecting high emotional arousal. It was characterized by high pitch, high loudness, a lowering of the jaw (what they called “mouth stretching”), and an expansion of the arms laterally. This cluster was most indicative of anger and joy among the 12 individual emotions that were analyzed. Scherer and Ellgring accounted for this profile as conveying a sense of urgency. Not only did we replicate this cluster in our dimensional analysis of the emotions, but we found a similar cluster when *characters* were organized along the dimension most conceptually similar to arousal, namely assertiveness. Scherer and Ellgring also observed something of the reverse cluster, what they called “multimodal resignation”. This would correspond to the low-assertiveness end of our character spectrum. All of these results support the contention that “quantity” (i.e., intensity) is more reliably encoded than “quality” (i.e., valence) when it comes to the conveyance of emotions and characters, both for the body^19^ and the voice^62^. This seems to be a general finding not only for production but for perception as well (e.g., Stevens et al., 2009)^63^. By contrast, our previous study of facial expression found stronger effects for character cooperativeness (quality) than assertiveness (quantity), consistent with the contention that the face is very good at representing emotional quality^64–66^. A comparative view of the expressive effectors suggests that the body might be most similar to the *voice* in its ability to represent emotional quantity, as compared to the face, which might be more specialized for representing emotional quality.

Our segmental results for characters are consistent with the single-marker results for emotions obtained by Dael, Mortillaro and Scherer^20^. Their analysis found that the three most salient dimensions of bodily expression were: (1) the location of the arms in relation to the body, similar to our horizontal arm and hand expansions, (2) the positioning of the upper body relative to the lower body, similar to our torso data, and (3) the positioning of the head relative to the body, similar to our head data. More specifically, our repeated-measures ANOVA results showed that character assertiveness was associated with expansion of the arms and hands, straightening of the torso, and raising of the head (see Supplementary Fig. 1). Comparing our findings to the studies of both Scherer and Ellgring^3^ and Dael, Mortillaro and Scherer^20^, we see parallel results for bodily expression between characters and emotions, something that we reported for the face in Berry and Brown^34^. We also see that a segmental approach to studying expression provides similar results to standard approaches that are based on single motion-capture markers alone. In our opinion, the segmental approach offers an advantage in that it opens up the analysis of expansion and contraction as salient descriptions of expression for the body and face^35^. This is particularly important in the analysis of bilateral movements, such as when the two arms move synchronously and symmetrically in opposite directions from the body core. Such a motion is more intuitively conceptualized as the expansion of a bilateral segment, rather than as two independent markers moving in opposite directions.

### Cross-modal correlations

Our previous study^34^ found bimodal correlations between jaw expansion and both vocal pitch and loudness for both character assertiveness and emotional arousal. These jaw/voice correlations turned out to be the strongest intermodal correlations in the present analysis. The current study added body correlations onto them, most notably raising of the head and expansion of the arms. In Fig. 5, we associated these two effects with different functional systems of expression, one for the vocal tract and the other that interfaces with the limbs.

1. The vocal tract system. The vocal tract system establishes the conditions for efficient vocal production, whether in the conveyance of emotions or in the production of communicative sounds like singing. There is strong anatomical, functional, and neural coupling among the effectors of this system^67–69^. The results showed that actors tended to both lower their jaw and raise their head in producing sounds that were loud and high-pitched. Both of these movements influence the shape of the vocal tract: jaw lowering enlarges the aperture of the vocal tract, while head raising straightens the curvature of the vocal tract. Both of these motions are common prescriptions in the training of professional singers to increase the resonance of the voice^70^. Jaw lowering is mediated by a series of jaw depressor muscles, which include the mylohyoid muscle, geniohyoid muscle, and the anterior belly of the digastric muscle^71, 72^. Such muscles not only control the jaw itself, but function additionally as extrinsic muscles of the larynx. Contraction of this group of muscles draws the mandible and hyoid bone together, simultaneously lowering the jaw and raising the larynx. The latter movement has a small effect on raising vocal pitch^73^. Hence, the anatomical coupling between head raising and jaw lowering works to promote efficient vocalization that is loud, high-pitched, and resonant in the conveyance of emotional arousal and—in the case of acting—character assertiveness as well.
2. The interface system. The other coupling with the voice and jaw observed in this study beyond the head was with the upper limbs, as shown by both horizontal and vertical expansions in the segments associated with the arms. This finding provides the strongest example of a body/face/voice triadic interface in our dataset. The spatial dimensions of body expansion have been well-analyzed in Laban’s^35^ detailed description of what he called the kinesphere of the body^74^. While Laban’s analysis spans the entire body, we focused our attention on the upper limbs alone, since this is the part of the body that is most commonly used in co-speech gesturing^6, 75^. The most novel finding of the correlation analysis was the observation of *a* *bimodal* *relationship* *between* *the* *arms* *and* *voice* as two mutually-reinforcing effector systems. The analysis showed that arm expansions were more strongly associated with loudness than with pitch, although both correlations were statistically significant.
  Music theorists describe pitch as occurring in a one-dimensional pitch space analogous to the vertical dimension of body space, such that pitch can rise and fall within this space^76^. A principal observation of the current study is that *rises* *in* *body* *space* *were* *mirrored* *by* *rises* *in* *pitch* *space.* In fact, the vertical dimension of arm movement showed slightly stronger correlations with pitch than did the horizontal dimension. This was reinforced by a raising of the head and a straightening of the torso. Another way of thinking about this phenomenon is that expansion of the limbs conveys emotional intensification in the visual-kinetic domain in a parallel manner to an increase in vocal pitch and loudness in the acoustic domain, in both cases increasing emotional quantity. This is therefore a clear example of multichannel reinforcement, one that resembles the parallels between pitch and spatial height in the domain of perception^77^.
  The limb/voice relationship is characterized not only by a similar dynamic when it comes to emotional intensification, but also by a similar precision in timing. It is perhaps no accident that we dance with our body, rather than with our face. The face seems to operate in a more static manner as a “shaping” system, analogous to a mask. This makes it ideally suited for static expression, such as in the case of visual art. The body too can function statically through the generation of static poses^14^. However, it more generally functions in a dynamic manner, which is useful for beating gestures, dancing, and the playing of musical instruments. When it comes to the face itself, the jaw is its most dynamic component, as seen in the mandibular articulatory movements that contribute to speech production and babbling^69^. Overall, the results suggest the possibility that there might be a stronger coupling between the upper limbs and the voice, as compared to either one in relation to the face. The limb/voice relationship merits further exploration.

### Implications for a theory of acting

Theatre as an artform in human cultures dates back to at least the ancient Greeks if not much earlier in indigenous theatrical traditions^27, 29^. However, there has been minimal study of the process of acting in the field of experimental psychology, despite the psychological richness of acting as a behavioral phenomenon^26, 78^. Instead, individual actors have been employed to generate prototypical stimuli for perceptual studies of human expression. This leaves unanswered the question of how actors create these expressions to begin with, as well as the variability with which this occurs across different performers. Acting theorists since the time of Aristotle have contrasted psychological (“inside-out”) and gestural (“outside-in”) approaches to the portrayal of fictional characters^25, 79^. Our work has focused on the gestural side of the actor’s method if only because of the great difficulties involved in elucidating psychological mechanisms of getting into character, short of using neuroimaging methods^80^. Hence, we have examined how actors externalize their representations of characters in terms of changes to their body gestures, facial expressions, and vocal prosodies. However, the nature of the present study—using stock characters in the absence of a dramatic context—may have favored the use of a gestural approach to portrayal. It is possible that the use of complex characters in dramatic contexts may have led to subtler effects than the ones we observed. There is a great need to expand the experimental approach to acting, not just because to its relevance to theatre, but because of the insights it brings to the study of self-processing, pretense, gesturing, and emotional expression^81^. Behavioral evidence demonstrates that assuming an expansive posture feeds back to make non-actors feel more powerful^82^. Such proprioceptive feedback might support the gestural methods that some actors use to portray characters^25^.

### Limitations

Limitations of this work include the relatively small numbers of characters and emotions that were examined in this study. In addition, all of the characters were basic, archetypal characters, rather than complex and/or more realistic characters, like Romeo or Juliet^83, 84^. Moreover, while assertiveness and cooperativeness have been effective at predicting expressive changes across the body, face, and voice in our studies, they are by no means the only personality traits that are relevant in describing characters. Other important traits include intelligence, extraversion, introversion, or even valence and arousal more directly. Extraversion/introversion has been used to describe social stereotypes through associative tasks^85^. Beyond personality, characters may also be described by the roles/functions they serve in the narrative^86, 87^. Next, the ecological validity of the work could be increased by having the actors do their performances in front of an audience. Likewise, the actors could be presented with the characters in advance of the experiment, allowing them to produce more rehearsed and polished performances. Finally, while we demonstrated parallels between character and emotion dimensions in our analysis, it would be useful to examine interactions between characters and emotions. A hero need not always be heroic. Depending on the character’s experiences and interaction partners, they may feel emotions like tenderness or sadness, or even experience a feeling of hopelessness and defeat at times. Characters are not monolithic entities with fixed traits, but instead people who experience varying emotional states across a story’s emotional arc and who manifest these emotional states gesturally with considerable variation.

## Conclusions

We have, to the best of our knowledge, carried out the first experimental study of the body correlates of character portrayal in professional actors, and found that character assertiveness was a better predictor of bodily expression than was character cooperativeness. We compared the body results with facial and vocal data for the same acting trials, and observed significant cross-modal correlations in the coding of assertiveness and the related emotion dimension of arousal. Part of this correlated expression emerged from a coupling of the effectors that support the conveyance of intensity in vocal expression, including head raising and jaw lowering. Another aspect resulted from a coupling of the vocal channel with the upper limbs, including the association of pitch rises with expansions in both the vertical and horizontal arm segments. Expansion of the limbs conveys emotional intensification in the visual-kinetic domain in a parallel manner to increases in vocal pitch and loudness in the acoustic domain, hence being a clear example of multichannel reinforcement. Overall, the results not only enlighten the multimodal nature of human communication, but provide new insights into the mechanisms of acting and the character/emotion relationship.

## Supplementary Information

Supplementary Information.

## Data Availability

All dependent variables (or measures) and independent variables (or predictors) that were used for analysis for this article’s target research questions have been reported in the Methods section. All exclusions or transformation of observations and their rational have been reported in the Methods section. The raw data for this study are available upon request by contacting the corresponding author.
