# Supplementary material for: Correlated expression of the body, face, and voice during character portrayal in actors
Source: Sci Rep. 2022 May 18;12:8253. doi: 10.1038/s41598-022-12184-7 (PMC9117657; doi:10.1038/s41598-022-12184-7)
Supplement: Supplementary file 1 — Supplementary Information. [file 41598_2022_12184_MOESM1_ESM.pdf]

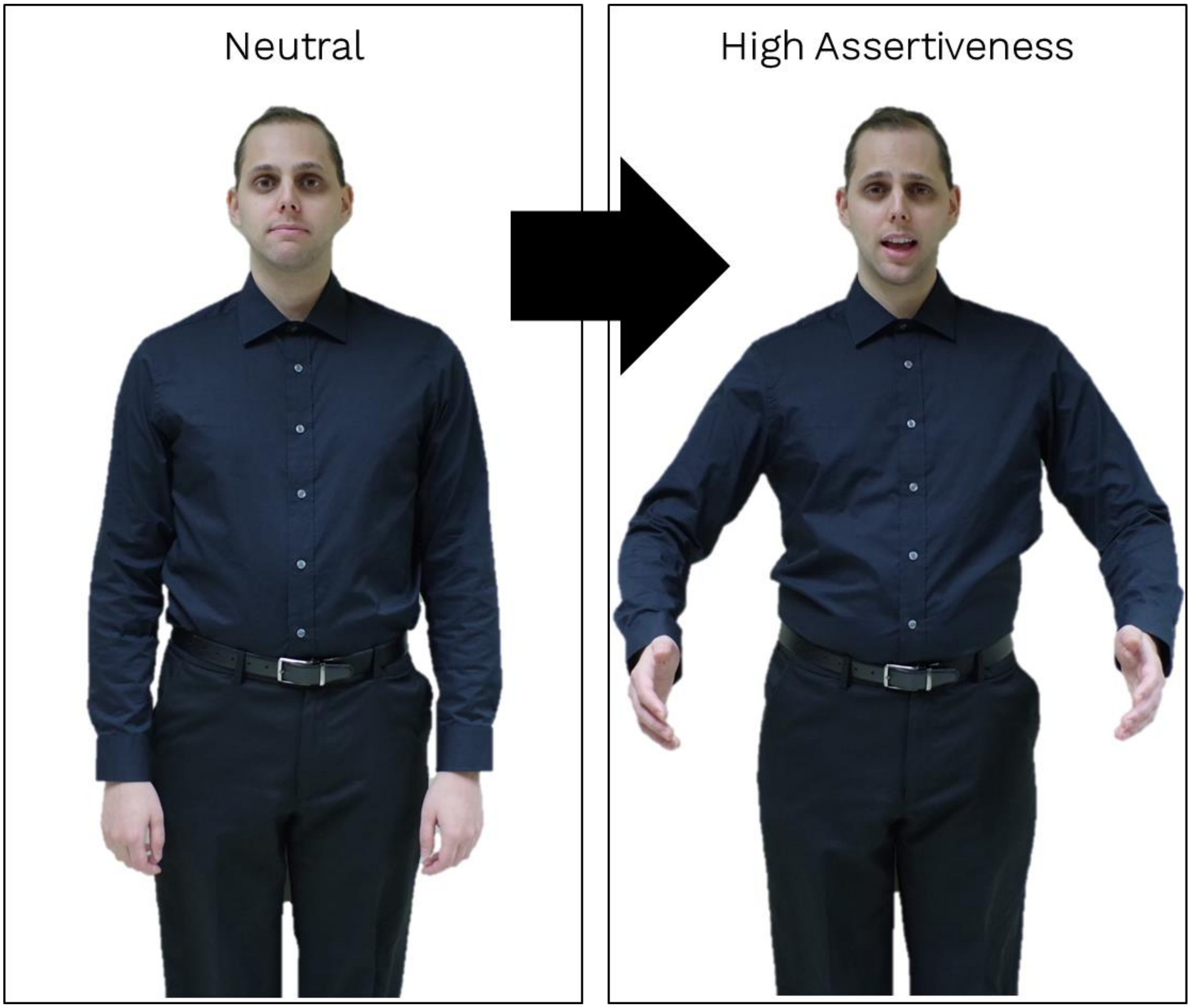

Supplementary Figure 1. A comparison of the neutral posture to the average body posture for high assertiveness for the 6 segments analyzed. Notable differences occur in the head (head raising), face (jaw lowering), and body (vertical and horizontal arm expansion).

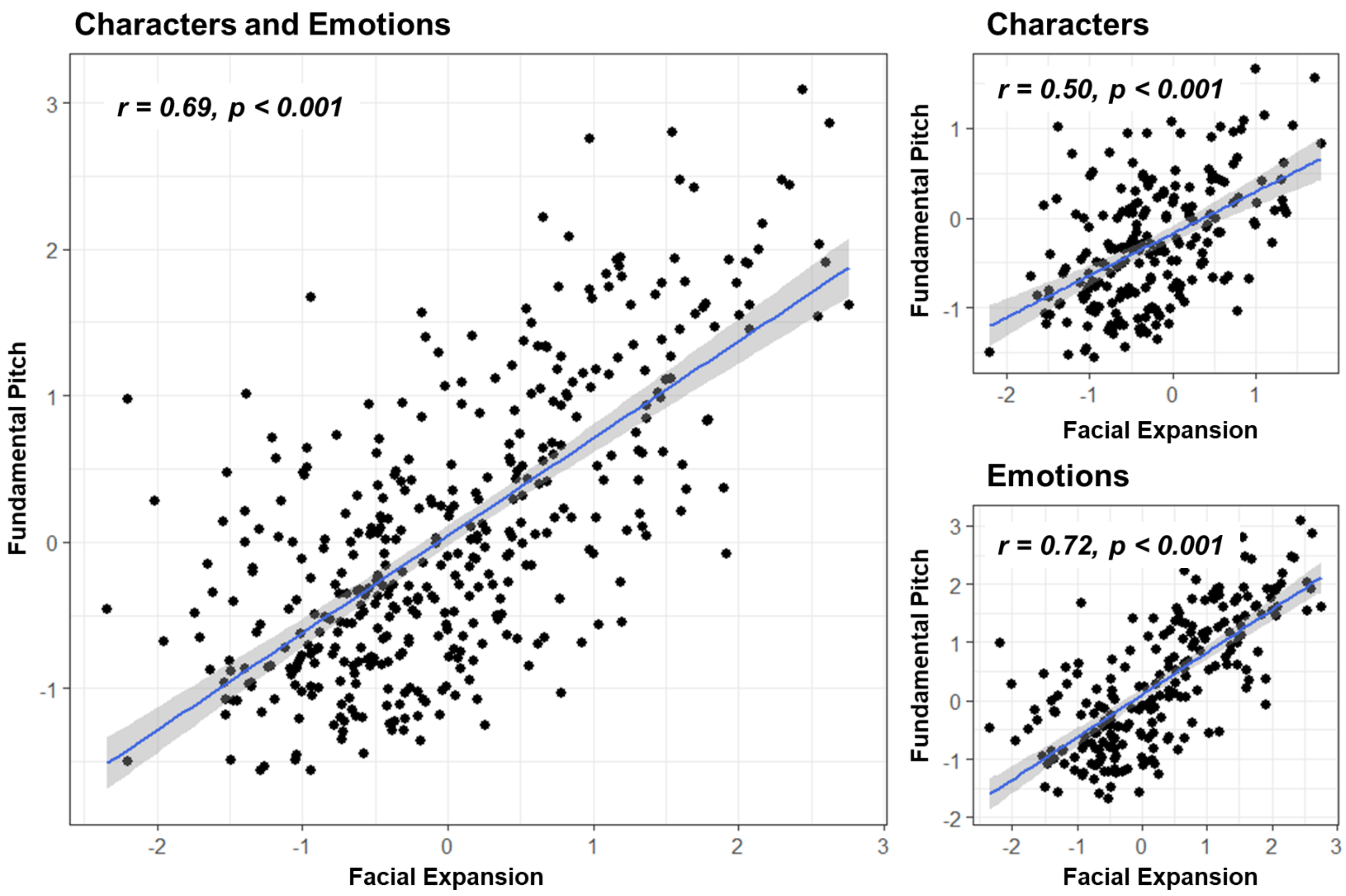

Supplementary Figure 2. Voice and face correlation for characters and emotions. The figure shows a linear regression and correlation of normalized pitch and jaw expansion for the combined character and emotion data (left panel), for the character data alone (top-right panel), and for the emotion data alone (bottom-right panel). Values are the percent change relative to the neutral emotion condition, which corrects for the diversity of body dimensions across participants, and z-transformed to account for scaling differences. The regression line is depicted in blue, while the shaded area indicates the standard error of the regression line. Pearson product-moment *r* values and significant *p values* are presented in the top left corner of each panel.

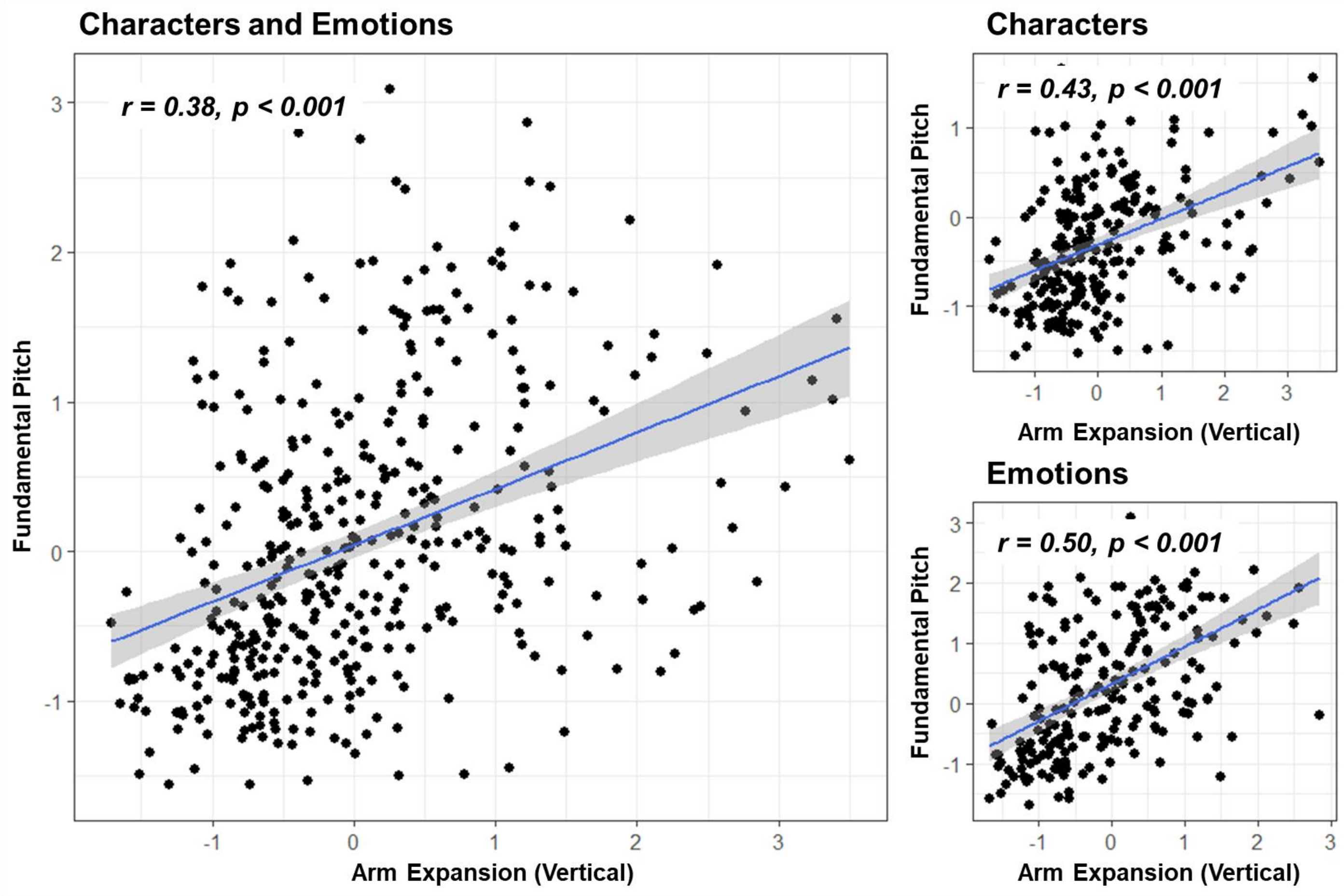

Supplementary Figure 3. Voice and body correlation for characters and emotions. The figure shows a linear regression and correlation of normalized pitch and vertical arm expansion for the combined character and emotion data (left panel), for the character data alone (top-right panel), and for the emotion data alone (bottom-right panel). Values are the percent change relative to the neutral emotion condition, which corrects for the diversity of body dimensions across participants, and z-transformed to account for scaling differences. The regression line is depicted in blue, while the shaded area indicates the standard error of the regression line. Pearson product-moment *r* values and significant *p values* are presented in the top left corner of each panel.

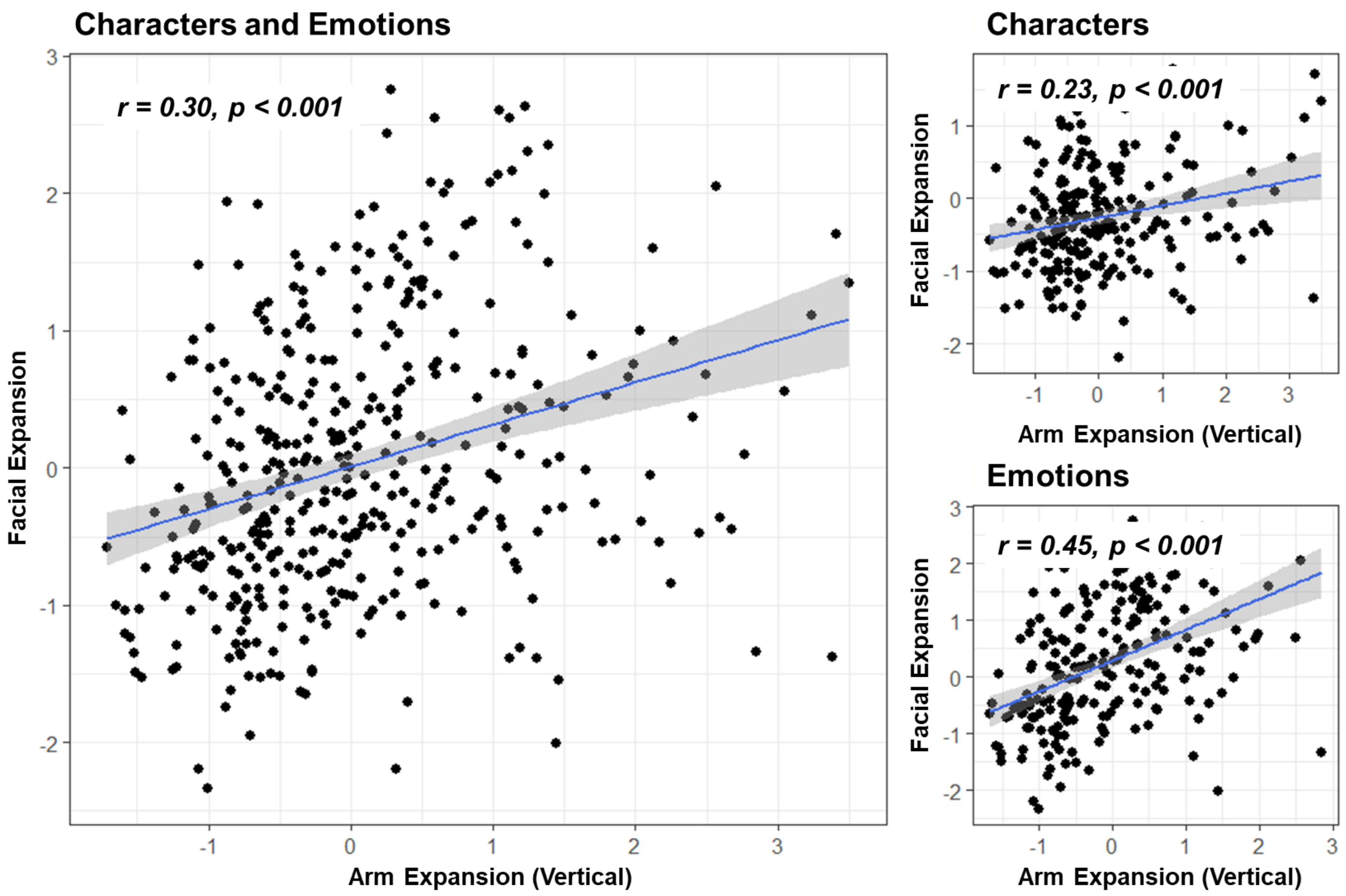

Supplementary Figure 4. Face and body correlation for characters and emotions. The figure shows a linear regression and correlation of jaw expansion and vertical arm expansion for the combined character and emotion data (left panel), for the character data alone (top-right panel), and for the emotion data alone (bottom-right panel). Values are the percent change relative to the neutral emotion condition, which corrects for the diversity of body dimensions across participants, and z-transformed to account for scaling differences. The regression line is depicted in blue, while the shaded area indicates the standard error of the regression line. Pearson product-moment *r* values and significant *p values* are presented in the top left corner of each panel.
